# Quality Improvement Project: RCPsych Portfolio Training for Psychiatry Trainees at East London NHS Foundation Trust

**DOI:** 10.1192/bjo.2025.10455

**Published:** 2025-06-20

**Authors:** Keerthi Vijayan, Kristen Hindley, Sen Kallumpuram, Aneeba Anwar, Natalie Ashburner

**Affiliations:** East London NHS Foundation Trust, Luton and Bedfordshire, United Kingdom

## Abstract

**Aims:** A questionnaire distributed to psychiatry trainees at the East London NHS Foundation Trust ascertained that, as of July 2024, trainees had not received formal training on navigating the Portfolio Online system. Additionally, there was no easily accessible document outlining key information for understanding the portfolio and the Annual Review of Competency Progression (ARCP) requirements.

This project aimed to enhance trainees’ understanding of the RCPsych Portfolio, improving their ability to navigate it efficiently. By addressing common challenges and providing clear guidance, the goal was to streamline the portfolio experience, enabling trainees to document their competencies and progress confidently.

**Methods:** An initial survey was conducted among trainees at the East London NHS Foundation Trust to assess their current training on portfolio navigation and ARCP requirements. Key findings included:

94.1% of participants had not received formal training on the RCPsych Portfolio.

64.7% were unclear about the contents of the Portfolio, despite available resources on the RCPsych website.

70.5% were unaware of all ARCP requirements.

100% of trainees felt that formal training on RCPsych Portfolio would be beneficial.

Based on these results, the following interventions were implemented:

Trainee Portfolio Handbook: A concise, user-friendly handbook was created, offering step-by-step instructions on key portfolio components and ARCP requirements for easy reference.

**Formal Training Sessions:** Structured, interactive sessions were organised to introduce trainees to the Portfolio and to clarify the ARCP expectations. These sessions are now incorporated into the local induction programme to ensure standardised training for all incoming trainees.

**Results:** Following the introduction of formal teaching and handbook distribution, feedback included:

100% of participants found the teaching sessions “extremely useful” and reported improved navigation of the portfolio.

100% respondents found the handbook very effective in improving their understanding of the portfolio.

86% found the handbook “extremely useful” in clarifying ARCP expectations.

The explanation of Placement-Specific Personal Development Plans was rated as particularly helpful.

Regular training sessions on RCPsych Portfolio are now being provided to all new psychiatry trainees at East London NHS Foundation Trust.

**Conclusion:** This Quality Improvement Project successfully enhanced the accessibility of resources and improved trainee comprehension of the RCPsych Portfolio and ARCP requirements. The positive feedback highlights the effectiveness of the handbook and training sessions. Additionally, the handbook has been submitted to the Royal College for review and potential inclusion in their official resources.

